# Patient-Clinician Sex and Race and/or Ethnicity Concordance and Adherence to Preventive Services Guidelines: MEPS 2018–2020

**DOI:** 10.1007/s11606-025-09631-2

**Published:** 2025-06-03

**Authors:** Alexis L. Green, Randy Le, Erik J. Rodriquez, Anna M. Nápoles, Eliseo J. Pérez-Stable, Paula D. Strassle

**Affiliations:** 1 Division of Intramural Research, National Institute On Minority Health and Health Disparities, National Institutes of Health, Bethesda, MD, USA; 2 Division of Intramural Research, National Heart, Lung, and Blood Institute, National Institutes of Health, Bethesda, MD, USA; 3 Department of Epidemiology and Biostatistics, University of Maryland, College Park, MD, USA

**Keywords:** concordance, race, ethnicity, sex, preventive services, cancer screening, vaccination

## Abstract

**BACKGROUND:**

Patient-clinician sex, racial, and ethnic concordance have been shown to improve healthcare utilization, but the impact of each on adherence to preventive services guidelines among specific populations remains unclear.

**OBJECTIVE:**

To estimate the association between patient-clinician sex and racial and/or ethnic concordance and adherence to preventive services guidelines.

**DESIGN:**

Cross-sectional study using nationally representative data from the Medical Expenditure Panel Survey (2018, 2020).

**PARTICIPANTS:**

Adults ≥ 18 years old who reported having a usual healthcare clinician. Adults who identified as multiracial, identified their clinician as being multiracial, or who did not report clinician sex, race, or ethnicity were excluded.

**MAIN MEASURES:**

Adherence to preventive services guidelines for influenza, pneumococcal, and shingles vaccines; breast, cervical, and colorectal cancer screening; and blood pressure and cholesterol screening. Predicted marginal prevalences and prevalence ratios were estimated using multivariable logistic regression, adjusting for sociodemographics, chronic conditions, and self-reported health status.

**KEY RESULTS:**

Females were less likely to report sex concordance compared to males (52.5% vs. 69.8%, *p* < 0.01). Among females, sex concordance increased influenza (PR = 1.08, 95% CI = 1.04–1.12), pneumococcal (PR = 1.06, 95% CI = 1.02–1.11), and shingles (PR = 1.09, 95% CI = 1.01–1.17) vaccination, as well as breast (PR = 1.06, 95% CI = 1.01–1.10), cervical (PR = 1.09, 95% CI = 1.05–1.13), and colorectal (PR = 1.07, 95% CI = 1.03–1.10) cancer screening, but not among males. Racial and/or ethnic concordance was low among American Indian and Alaska Native, Black, Latino, and Native Hawaiian and Pacific Islander patients (< 25%) and was not associated with adherence in preventive services.

**CONCLUSIONS:**

Females with female clinicians are more likely to adhere to preventive services guidelines. Racial and/or ethnic concordance was not associated with adherence to preventive services guidelines, but racial and/or ethnic concordance was low among non-White patients. Sex and racial and/or ethnic concordance may be a powerful tool for increasing preventive services utilization, but increased racial and/or ethnic concordance is needed to reach more definitive conclusions.

## INTRODUCTION

Preventive services, like cancer screenings and vaccinations, are powerful tools for improving health and preventing disease; however, fewer than 10% of US adults receive all appropriate, high-priority clinical preventive services.^1,2^ Racial and/or ethnic differences in adherence to preventive service guidelines have been well described, with Asian, Black, and Latino adults having lower adherence rates compared to White adults.^3–9^ Sex differences in preventive services are mixed, with some studies documenting that males are more likely to utilize preventive services, while others show females are more likely or no sex differences.^9–11^

There is some evidence that increasing the diversity of primary clinicians could improve preventive services utilization. Sex concordance, defined as when a patient and clinician are either both male or both female, has been associated with improved surgical outcomes,^12^ myocardial infarction survival in female patients,^13^ and patient-centered care.^14^ Sex discordance has also been shown to negatively affect female patients, as male clinicians have been found to be more likely to have negative perceptions of their female patients and assume their conditions are less severe.^15^ Patient-clinician racial and/or ethnic concordance, defined as when the patient and healthcare clinician share the same racial and/or ethnic identity, has been associated with increased in-person health visits,^16^ decreased newborn mortality,^17^ decreased emergency department use,^18^ lower total healthcare expenditures,^18^ and improved patient satisfaction.^19^ Studies on patient-clinician racial and/or ethnic concordance and preventive services have been mixed.^20,21^

Despite these generally positive findings of sex and racial and/or ethnic concordance on healthcare utilization and outcomes, relatively little is known about the impact of patient-clinician concordance on preventive services. Thus, the purpose of this study was to assess (1) the association between patient-clinician sex concordance and adherence to preventive services guidelines, overall and within females and males, and (2) the association between patient-clinician racial and/or ethnic concordance and adherence to preventive services guidelines, overall and within each racial and/or ethnic patient population. Given the relatively low rates of adherence to several preventive services guidelines in the USA and prevalent racial and/or ethnic differences in infectious and chronic diseases, understanding how patient-clinician concordance may improve uptake of preventive services guidelines, especially among female and non-White patients, is critical.

## METHODS

### Data Source and Study Population

This cross-sectional study utilized data from the 2018 and 2020 Medical Expenditure Panel Survey (MEPS), a nationally representative survey of healthcare cost, utilization, and health insurance coverage in the USA. A detailed description of the MEPS study design and survey instruments can be found online (https://meps.ahrq.gov/mepsweb/survey_comp/survey.jsp). In short, we utilized the household component of the MEPS, which collects data from a sample of families and individuals drawn from a nationally representative subsample of households that participated in the National Health Interview Survey (conducted by the National Center for Health Statistics). In prior iterations of MEPS, households were interviewed five times over a 2-year period, but in the spring of 2020, in-person data collection was limited due to the COVID-19 pandemic. The MEPS survey team adapted data collection by overlapping three simultaneous panels of data collection in the spring of 2020, compared to the usual two-panel overlap. Some in-person interviews were also moved to telephone surveys.

For this analysis, all adults ≥18 years old who completed the 2018 or 2020 MEPS survey were eligible for inclusion (*n*=44,464). Participants were excluded if they identified as multiracial (*n*=1030) or if they reported that they did not have a usual healthcare clinician (*n*=12,848). Participants were also excluded if clinician race and/or ethnicity was reported as multiracial or missing (*n*=10,160) or if clinician sex was missing (*n*=3). Our final unweighted sample size was 20,423 adults.

### Adherence to Preventive Services Recommendations

For this analysis, adherence to eight preventive services recommended by the United States Preventive Services Task Force (USPSTF) or Advisory Committee on Immunization Practices (ACIP) was assessed: influenza vaccine, pneumococcal vaccine, shingles vaccine, breast cancer screening (female only), cervical cancer screening (female only), colorectal cancer screening, blood pressure screening, and cholesterol screening. Participants were only asked questions about preventive services where they fell within the recommended age group (and sex) based on published guidelines (e.g., breast cancer screening is recommended for female adults ages 50–74, so females outside of that age range and all males skipped those questions) (Supplemental Table 1). Participants also skipped questions if they reported having a contraindication for the preventive service; for example, female participants with a history of breast cancer or mastectomy were not asked about routine breast cancer screening even if they fell within the age range for screening.

Adherence to colorectal cancer screening recommendations was captured using three questions, given that multiple screening tools are available (colonoscopy, sigmoidoscopy, fecal occult blood test), and each has its own recommended timeframe. Participants were classified as being adherent if they met the guidelines for at least one of the screening tools.

### Patient-Clinician Race and/or Ethnicity and Sex Concordance

Race and/or ethnicity concordance and sex concordance variables were created using participant-reported participant and clinician demographics. Participants were asked about their ethnicity (“Are you Hispanic, Latino, or of Spanish origin?”) and their race (“What is your race?”) separately as part of the survey and then combined into a single variable. Individuals were categorized as Hispanic or Latino (Latino) regardless of their response to the race question. For non-Hispanic or non-Latino participants, race and/or ethnicity was categorized as American Indian or Alaska Native (AIAN), Asian (including Asian Indian, Chinese, Filipino, Japanese, Korean, Vietnamese, and other Asian), Black or African American (Black), Native Hawaiian or Pacific Islander (NHPI, including Native Hawaiian, Guamanian or Chamorro, Samoan, and Other Pacific Islander), and White. As mentioned above, participants who identified as multiracial or other race (and not Latino) were excluded due to our inability to determine concordance with their clinician.

Participants were similarly asked about their clinician race and/or ethnicity in separate questions and then categorized using the same criteria described above. Patient-clinician racial and/or ethnic concordance was defined as when the participant’s race and/or ethnicity matched the clinician’s race and/or ethnicity (as reported by the participant). Participants with clinicians identified as multiracial were also excluded.

Patient-clinician sex concordance was similarly defined as when a participant’s sex matched their reported clinician’s sex. Participants were asked “What is your sex?” (male or female) and “Is [your clinician] male or female?”

### Statistical Analysis

Multivariable logistic regression was used to model the association between patient-clinician concordance and adherence to each preventive services guideline as well as calculate the predicted marginal prevalence of adherence and prevalence ratios,^22^ comparing adherence between those with and without patient-clinician concordance; sex concordance and racial and/or ethnic concordance were evaluated separately. Models adjusted for patient sex, race and/or ethnicity, age, educational attainment (less than high school graduate, high school graduate/GED, post-secondary degree), annual family income, insurance type (any private, public only, uninsured), self-reported chronic conditions (heart disease or stroke, diabetes, asthma, and cancer), self-reported general health, and survey year. Sex concordance models were also adjusted for clinician race and/or ethnicity, and race and/or ethnicity concordance models were adjusted for clinician sex. Age and family income were modeled as restricted quadratic splines. Self-reported overall health was captured with the question, “In general, would you say your health is…” with answer choices of excellent, very good, good, fair, or poor.

We used interaction terms and population-specific models to estimate the association of sex concordance among males and females separately (e.g., the association between sex concordance on influenza vaccination among female patients), as well as the association between racial and/or ethnic concordance among each racial and/or ethnic patient population. Because results from both approaches were similar, only results from the population-specific models are reported. Due to small sample sizes and very low prevalence of patient-clinician race and/or ethnicity concordance, AIAN and NHPI population-specific estimates could not be generated.

We also conducted a sensitivity analysis where race and/or ethnicity concordance models were restricted to non-White participants, as well as where race and/or ethnicity concordance was reclassified from yes/no: (1) yes, (2) no, but clinician is non-White, and (3) no and clinician is White.

Analyses were performed using SAS version 9.4 (SAS Inc, Cary, NC) and SUDAAN Release 11.0.4 (RTI International, Research Triangle Park, NC), were weighted, and were accounted for the complex sample design used in MEPS. This study was conducted in accordance with STROBE guidelines.

## RESULTS

**Figure 1:**
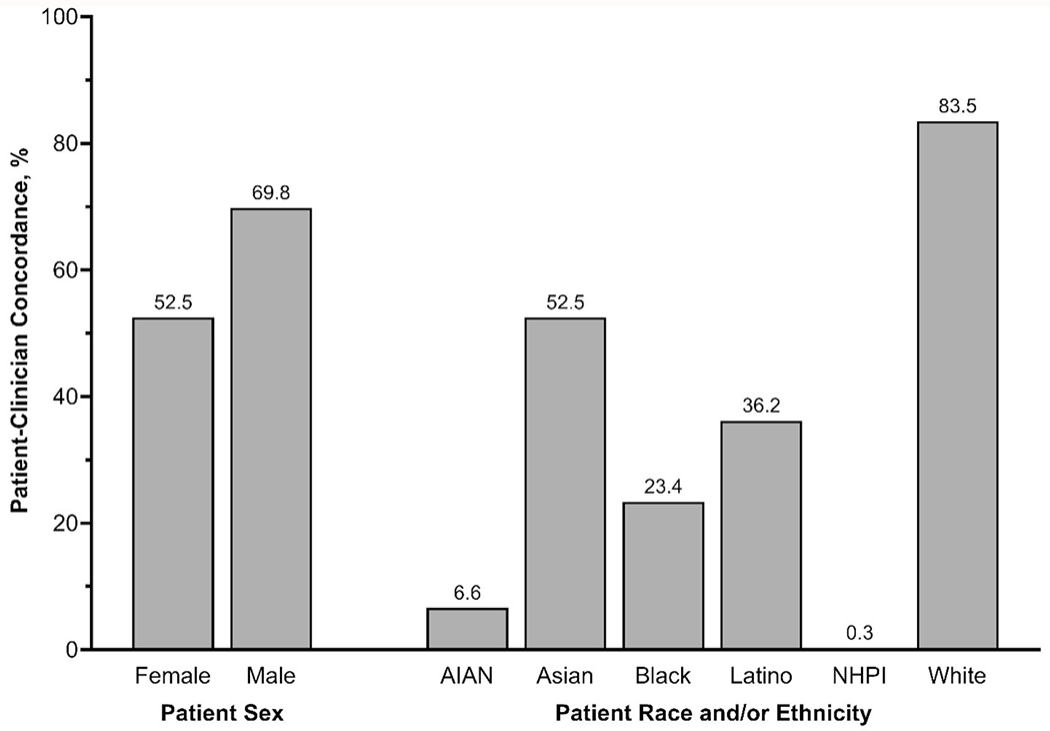
Prevalence of patient-clinician sex concordance, stratified by sex, and patient-clinician race and/or ethnicity concordance, stratified by patient race and/or ethnicity. AIAN, American Indian or Alaska Native; NHPI, Native Hawaiian or Pacific Islander.

Overall, 60.2% of MEPS participants with a usual primary clinician had sex concordance with their healthcare clinician, and 69.1% had racial and/or ethnic concordance. Female participants were less likely to report sex concordance with their clinician, compared to male participants (52.5% vs. 69.8%, *p*<0.0001) (Fig. 1 and Supplemental Table 2). Female participants also had slightly less race and/or ethnicity concordance with their clinician, compared to males (68.3% vs. 70.0%, *p*=0.02).

Most White participants reported racial and/or ethnic concordance with their clinician (83.5%), followed by Asian participants (52.3%) (Fig. 1 and Supplemental Table 2). Less than half of Black (23.4%) and Latino (36.2%) participants reported racial and/or ethnic concordance with their clinician, and concordance was very low among AIAN (6.6%) and NHPI (0.3%) participants. Compared to White adults, all other racial and/or ethnic populations had lower racial and/ or ethnic patient-clinician concordance (*p*<0.0001 for all). Latino participants were also less likely to have sex concordance with their clinician, compared to White participants (57.1% vs. 60.5%, *p*=0.008), but otherwise no differences in patient-clinician sex concordance were observed across race and/or ethnicity.

Overall, adherence to preventive services guidelines was similar between males and females, with females being slightly more likely to be adherent (0.4% to 3.5% difference) (Supplemental Table 3). Racial and/or ethnic differences in adherence were observed, with Asian and White participants generally exhibiting higher adherence to vaccinations and cancer screenings (Supplemental Table 4). Adherence to blood pressure and cholesterol screenings was high across all race and/or ethnicity populations (>90.0% for all).

### Association Between Patient-Clinician Sex Concordance and Preventive Services

Overall, patient-clinician sex concordance was associated with modest increases in vaccination adherence (prevalence ratios [PRs]=1.03 to 1.05), but no significant difference was observed for colorectal cancer screening (PR=1.02, 95% CI=0.99–1.04), blood pressure screening (PR=1.01, 95% CI=1.00–1.01), or cholesterol screening (PR=1.00, 95% CI=0.99–1.01) (Supplemental Table 5).

However, when analyses were stratified by sex, different relationships were observed for females and males (Table 1). Among females, patient-clinician sex concordance was associated with an increase in influenza (PR=1.08, 95% CI=1.04–1.12), shingles (PR=1.09, 95% CI=1.01–1.17), and pneumococcal (PR=1.06, 95% CI=1.02–1.11) vaccine adherence, as well as breast cancer screening (PR=1.06, 95% CI=1.02–1.10), cervical cancer screening (PR=1.09, 95% CI=1.05–1.13), and colorectal cancer screening (PR=1.07, 95% CI=1.02–1.09). Among males, patient-clinician sex concordance appeared to have no meaningful impact (PRs=0.98 to 1.02).

### Association Between Patient-Clinician Racial and/or Ethnic Concordance and Preventive Services (Main and Sensitivity Analyses)

Racial and/or ethnic concordance was not significantly associated with adherence to preventive services recommendations, overall or among any specific racial and/or ethnic populations (Table 2 and Supplemental Tables 6–7). When patient-clinician race and/or ethnicity discordance was stratified into non-White and White clinicians, similar results were seen (Supplemental Table 9).

## DISCUSSION

In a national sample of US households, we found that in 2018 and 2020, only half of female adults had sex concordance with their primary clinician, compared to over two-thirds of male adults. Patient-clinician race and/or ethnicity concordance was low (<25%) among AIAN, Black, Latino, and NHPI adults. Patient-clinician sex concordance had minimal impact on adherence to preventive services guidelines among males but was associated with an almost 10% increase in adherence among females. Racial and/or ethnic concordance appeared to have minimal effect on adherence to preventive services guidelines. Given the importance of preventive services as a public health tool to reduce disease incidence and mortality, even relatively modest increases in uptake due to patient-clinician concordance could have large positive effects on the prevalence of infectious and chronic diseases in the USA.

Our findings that patient-clinician sex concordance among females increased adherence to preventive services guidelines are consistent with previous studies, which also found increased vaccine uptake and cancer screening rates among female adults with female clinicians.^20,23,24^ Female patients with female clinicians have reported better relationships, communication, and patient satisfaction in gynecology,^25,26^ so it is possible that these associations are partially driven by female patients having greater trust in female physicians, especially since breast and cervical cancer screening fall under women’s healthcare. It is also possible that females able to seek out sex-concordant clinicians have better access to care, although we were able to adjust for several markers of healthcare access (e.g., household income, insurance) in our analysis.

Previous studies have found mixed effects of patient-clinician racial and/or ethnic concordance on adherence to preventive services guidelines, with some studies finding concordance improves utilization and others finding no meaningful effect.^20,21^ It is unclear why patient-clinician race and/or ethnicity concordance does not have a larger, positive impact on adherence to preventive guidelines since racial and/or ethnic concordance has been consistently associated with better patient experiences among Asian, Black, and Latino adults,^16,19,27,28^ and better patient experiences have been linked to higher utilization of preventive services.^29–31^ Counties with more Black clinicians have also been found to have increased life expectancy and lower mortality for Black populations, which could in part be due to higher patient-clinician race and/or ethnicity concordance.^32^ While we found that patient-clinician racial and/or ethnic concordance was not associated with preventive services overall or within specific racial and/or ethnic populations, race and/or ethnicity concordance was also low for AIAN, Black, Latino, and NHPI populations.

Cultural and language barriers for patients, which disproportionately affect non-White populations, are also important to address in the healthcare system. Language concordance, particularly for Spanish-speaking communities, improves healthcare outcomes for people who historically have been underserved by the US healthcare system.^33,34^ Limited English proficiency (LEP) has also been associated with lower adherence to preventive services,^35^ and access to professional medical interpretation services has been shown to improve healthcare utilization (including preventive services) among adults with LEP.^36^ It is possible that patient-clinician concordance across culture and language may play a bigger role than concordance across race and/or ethnicity alone, and could explain the mixed findings for racial and/or ethnic concordance on preventive services and other healthcare utilization. Future studies should incorporate other forms of patient-clinician concordance, like culture and language, as well as look at intersectional concordance across race and/or ethnicity, sex, culture, and language to better assess the role culture may play.

Our findings and others also show that patient-clinician concordance for AIAN, Black, Latino, and NHPI patients is low, largely due to the lack of diversity in the healthcare workforce. Medical school admissions are one area in which reform could improve representation and patient-clinician concordance.^37,38^ Removing barriers, like the cost of preparing for and attending medical school and insufficient counseling or mentorship prior to and during medical school, could increase the number of students pursuing and completing a medical education from these communities.^39,40^

This study has a few limitations. First, we used self-reported adherence to preventive services guidelines, which is vulnerable to recall bias, particularly for screenings that only need to occur every 5 or 10 years, as well as social desirability bias. Second, we had to limit our analysis to adults who reported having a usual primary clinician, which also increases adherence to preventive services guidelines.^41^ Several of our preventive service adherence rates were higher than national estimates, likely due to this restriction (e.g., influenza vaccination among our sample was 56.6% compared to the national estimate in 2020 of 50.2%).^42^ As such, these results may not generalize to adults without a place for usual healthcare. We also assessed sex and race and/or ethnicity concordance separately, but there are important intersections that exist across sex, race, and ethnicity that should be assessed to better understand concordance and preventive services. We also excluded multiracial adults when assessing racial and/or ethnic concordance, and results may not generalize to individuals who identify as multiracial or have multiracial physicians. Finally, a person’s decision to follow preventive services guidelines is influenced by many factors, and while we were able to control for sociodemographic and health-related risk factors, some unmeasured confounding likely exists.

This study also had several strengths. First, we utilized data from 2018 and 2020, which makes this the most recent assessment of patient-clinician concordance and adherence to preventive services guidelines. Given recent changes to the healthcare system, including the passage of the Affordable Care Act, which expanded health insurance coverage and required insurance plans to cover the costs of preventive services recommended by USPSTF and ACIP, updated assessments were needed. We are also one of the first studies to include AIAN and NHPI populations, although we were unable to include them in our race- and/or ethnicity-stratified analyses due to their low patient-clinician concordance rates. It is possible that increasing the representation of these populations in the healthcare system could have substantial effects, given how few AIAN and NHPI adults are treated by clinicians of the same race and/or ethnicity. We were also the first study to assess whether discordant, but non-White clinicians were associated with increased adherence to preventive services guidelines among non-White patient populations.

In conclusion, we found that patient-clinician sex concordance increased adherence to vaccination and cancer screening guidelines by almost 10% among females but had limited impact for males. Additional research is needed on the impact of patient-clinician racial and/or ethnic concordance, given the relatively low rates of concordance among non-White patients. Patient-clinician sex and racial and/or ethnic concordance could be an important tool for improving healthcare utilization and patient outcomes. Medical schools and health systems employers should focus on training, recruiting, and retaining health clinicians who are underrepresented in medicine.

## Supplementary Material

Supplementary Materials

The online version contains supplementary material available at https://doi.org/10.1007/s11606-025-09631-2.

## Figures and Tables

**Table 1:** Prevalence of Adherence to Preventive Services Guidelines Among Those With and Without Patient-Clinician Sex Concordance, and Association Between Patient-Clinician Sex Concordance and Preventive Services, Stratified by Patient Sex

	Female			Male		
	Sex concordance^a^	Sex discordance^a^	PR (95% CI)^a^	Sex concordance^a^	Sex discordance^a^	PR (95% CI)^a^
Vaccinations						
Influenza	60.3%	55.8%	1.08 (1.04, 1.12)	54.4%	55.5%	0.98 (0.93, 1.03)
Pneumococcal	79.1%	74.3%	1.06 (1.02, 1.11)	74.3%	71.4%	1.04 (0.98, 1.11)
Shingles	53.9%	49.6%	1.09 (1.01, 1.17)	51.2%	50.1%	1.02 (0.93, 1.12)
Cancer screening						
Breast	85.2%	80.5%	1.06 (1.02, 1.10)	–	–	N/A^b^
Cervical	87.0%	79.8%	1.09 (1.05, 1.13)	–	–	N/A^b^
Colorectal	81.0%	76.6%	1.06 (1.02, 1.09)	77.9%	79.3%	0.98 (0.94, 1.02)
Other screenings						
Blood pressure	97.2%	96.3%	1.00 (0.99, 1.01)	95.3%	95.5%	1.01 (1.00, 1.02)
Cholesterol	93.8%	91.6%	0.98 (0.96, 1.00)	91.9%	93.7%	1.02 (1.01, 1.04)

Abbreviations: *PR*, prevalence ratio; *CI*, confidence interval; *N/A*, not analyzable

a Prevalence ratio (PR) is the ratio of predicted marginal proportions calculated from the logistic regression model, adjusted for patient sex, race and/or ethnicity, age (treated as a restricted quadratic spline), highest education, family income (treated as a restricted quadratic spline), insurance status, self-reported chronic conditions (heart disease or stroke, diabetes, asthma, and cancer), self-reported general health, survey year, and clinician race and/or ethnicity

b Breast and cervical cancer screening guidelines only apply to females

**Table 2:** Association Between Patient-Clinician Race and/or Ethnicity Concordance and Adherence to Preventive Services Guidelines, Stratified by Patient Race and/or Ethnicity

	Asian PR (95% CI)^a^	Black PR (95% CI)^a^	Latino PR (95% CI)^a^	White PR (95% CI)^a^
Vaccinations				
Influenza	0.87 (0.71, 1.05)	0.95 (0.83, 1.10)	0.87 (0.71, 1.05)	0.95 (0.83, 1.10)
Pneumococcal	1.09 (0.80, 1.47)	0.79 (0.66, 0.94)	0.86 (0.72, 1.04)	0.97 (0.92, 1.03)
Shingles	0.72 (0.44, 1.20)	1.09 (0.80, 1.49)	0.77 (0.59, 1.00)	0.98 (0.90, 1.07)
Cancer screening				
Breast^b^	0.98 (0.80, 1.19)	1.00 (0.92, 1.10)	1.05 (0.95, 1.15)	0.97 (0.91, 1.03)
Cervical^b^	0.85 (0.71, 1.02)	1.03 (0.91, 1.17)	0.99 (0.90, 1.09)	1.04 (0.98, 1.09)
Colorectal	1.12 (0.91, 1.37)	0.90 (0.80, 1.02)	0.99 (0.88, 1.11)	0.99 (0.95, 1.04)
Other screenings				
Blood pressure	1.02 (0.93, 1.12)	1.01 (0.98, 1.04)	1.00 (0.97, 1.02)	1.00 (0.99, 1.01)
Cholesterol	1.07 (0.98, 1.18)	0.99 (0.95, 1.04)	1.02 (0.99, 1.07)	0.98 (0.96, 1.01)

Abbreviations: *PR*, prevalence ratio; *CI*, confidence interval

Due to small population sizes, AIAN and NHPI patients were excluded from the analysis

a Prevalence ratio (PR) is the ratio of predicted marginal proportions calculated from the logistic regression model, adjusted for patient sex, race and/or ethnicity, age (treated as a restricted quadratic spline), highest education, family income (treated as a restricted quadratic spline), insurance status, self-reported chronic conditions (heart disease or stroke, diabetes, asthma, and cancer), self-reported general health, survey year, and clinician sex

b Among females only

## Data Availability

The Medical Expenditure Panel Survey (MEPS) is a publicly available dataset, which can be accessed through the Agency for Healthcare Research and Quality at https://meps.ahrq.gov/mepsweb/data_stats/download_data_files.jsp. Computing code is available upon reasonable request to the corresponding author (P. Strassle).
