# Supplementary material for: Patient-Clinician Sex and Race and/or Ethnicity Concordance and Adherence to Preventive Services Guidelines: MEPS 2018–2020
Source: J Gen Intern Med. 2025 Jun 3;41(3):767–74. doi: 10.1007/s11606-025-09631-2 (PMC12332972; doi:10.1007/s11606-025-09631-2)
Supplement: Supplementary file 1 — Supplementary file1 (DOCX 56 KB) [file 11606_2025_9631_MOESM1_ESM.docx]

**Supplemental Appendix**

| **Supplemental Table 1**. Preventive services guidelines recommended by the U.S. Preventive Services Task Force (USPSTF) and the CDC Advisory Committee on Immunization Practices (ACIP) | | |
| --- | --- | --- |
|  | **Population^a^** | **Frequency** |
| **Vaccinations** |  |  |
| Influenza | All adults | Yearly |
| Pneumococcal^b^ | All adults 65+ | Once after 65 |
| Shingles | All adults 65+ | Once after 65 |
| **Cancer Screening** |  |  |
| Breast | Females 50-74 | 2 years |
| Cervical^c^ | Females 35-64 | 5 years |
| Colorectal | All adults 50-74 | Varies by test^d^ |
| **Other Screenings** |  |  |
| Blood pressure | All adults 35+ | 2 years |
| Cholesterol | All adults 35-74 | 5 years |
| ^a^ Unless contraindicated or inappropriate (e.g., allergy to vaccination contents, previous hysterectomy or mastectomy).  ^b^ In 2024, ACIP recommended lowering age of pneumococcal vaccination from 65 to 50 years old  ^c^ Pap or HPV testing in the past 5 years  ^d^ Every 1, 5, or 10 years based on screening test (fecal occult blood test: 1 year; flexible sigmoidoscopy: 5 years; colonoscopy: 10 years) | | |

**Supplemental Table 2.** Prevalence of patient-clinician sex and racial and/or ethnic concordance, among participants who reported having a usual clinician, stratified by patient sociodemographics. Results are weighted to be nationally representative and account for MEPS complex sample design.

|  | **Sex**  **concordance** | |  | **Racial and/or ethnic concordance** | |
| --- | --- | --- | --- | --- | --- |
|  |  | **p-value** |  |  | **p-value** |
| **Overall** | 60.2% | – |  | 69.1% | – |
| **Sex** |  |  |  |  |  |
| Female | 52.5% | <0.0001 |  | 68.3% | 0.02 |
| Male | 69.8% | ref |  | 70.0% | ref |
| **Race and/or ethnicity** |  |  |  |  |  |
| AIAN | 58.9% | 0.81 |  | 6.6% | <0.0001 |
| Asian | 63.8% | 0.09 |  | 52.3% | <0.0001 |
| Black | 60.1% | 0.73 |  | 23.4% | <0.0001 |
| Latino | 57.1% | 0.008 |  | 36.2% | <0.0001 |
| NHPI | 60.4% | 0.99 |  | 0.3% | <0.0001 |
| White | 60.5% | ref |  | 83.5% | ref |
| **Age group** |  |  |  |  |  |
| 18-34 years old | 61.9% | 0.93 |  | 65.2% | 0.88 |
| 35-49 years old | 61.8% | ref |  | 64.9% | ref |
| 50-64 years old | 60.1% | 0.12 |  | 70.2% | 0.0001 |
| ≥65 years old | 58.3% | 0.0008 |  | 72.8% | <0.0001 |
| **Educational attainment** |  |  |  |  |  |
| No degree | 57.1% | <0.0001 |  | 62.4% | <0.0001 |
| High school graduate/GED | 58.0% | <0.0001 |  | 68.4% | 0.01 |
| Postsecondary degree | 62.7% | ref |  | 70.9% | ref |
| **Annual family income** |  |  |  |  |  |
| <$20,000 | 55.1% | <0.0001 |  | 62.0% | <0.0001 |
| $20,000-$59,999 | 58.5% | <0.0001 |  | 68.5% | 0.04 |
| $60,000-$99,999 | 60.3% | 0.006 |  | 70.2% | 0.52 |
| ≥$100,000 | 63.1% | ref |  | 71.0% | ref |
| **Health insurance** |  |  |  |  |  |
| Any private | 61.7% | ref |  | 70.3% | ref |
| Public only | 56.2% | <0.0001 |  | 66.0% | 0.0002 |
| Uninsured | 57.3% | 0.07 |  | 65.6% | 0.08 |
| **Self-reported health status** |  |  |  |  |  |
| Excellent | 62.5% | ref |  | 69.3% | ref |
| Very good | 61.1% | 0.27 |  | 71.5% | 0.09 |
| Good | 59.1% | 0.008 |  | 67.6% | 0.22 |
| Fair/poor | 56.9% | <0.0001 |  | 65.5% | 0.01 |

AIAN=American Indian or Alaska Native; NHPI=Native Hawaiian or Pacific Islander

**Supplemental Table 3.** Prevalence of preventive services among participants who reported having a usual clinician, overall and stratified by sex. Results are weighted to be nationally representative and account for MEPS complex sample design.

|  | **Overall** | **Female** | **Male** |
| --- | --- | --- | --- |
| **Vaccinations** |  |  |  |
| Influenza | 56.6% | 58.2% | 54.7% |
| Pneumococcal | 75.2% | 76.5% | 73.5% |
| Shingles | 51.3% | 51.6% | 50.9% |
| **Cancer Screening** |  |  |  |
| Breast | N/A^a^ | 82.8% | N/A^a^ |
| Cervical | N/A^a^ | 83.7% | N/A^a^ |
| Colorectal | 78.6% | 78.9% | 78.3% |
| **Other Screenings** |  |  |  |
| Blood pressure | 96.3% | 96.8% | 95.8% |
| Cholesterol | 92.6% | 92.8% | 92.4% |
| ^a^ Breast and cervical cancer screening guidelines only apply to females and was only analyzed in sex-stratified analyses | | | |

**Supplemental Table 4.** Prevalence of preventive services among participants who reported having a usual clinician, overall and stratified by race and/or ethnicity. Results are weighted to be nationally representative and account for MEPS complex sample design

|  | **Overall** | **AIAN** | **Asian** | **Black** | **Latino** | **NHPI** | **White** |
| --- | --- | --- | --- | --- | --- | --- | --- |
| **Vaccinations** |  |  |  |  |  |  |  |
| Influenza | 56.6% | 57.8% | 60.5% | 43.4% | 46.1% | 57.3% | 59.8% |
| Pneumococcal | 75.2% | 73.1% | 64.0% | 63.3% | 61.1% | 63.1% | 78.1% |
| Shingles | 51.3% | 39.6% | 42.9% | 32.1% | 33.1% | 58.6% | 54.7% |
| **Cancer Screening** |  |  |  |  |  |  |  |
| Breast^a^ | 82.8% | 57.5% | 84.6% | 84.3% | 82.6% | 77.0% | 82.8% |
| Cervical^a^ | 83.7% | 95.6% | 74.8% | 75.5% | 86.0% | 75.3% | 81.8% |
| Colorectal | 78.6% | 76.8% | 74.1% | 77.3% | 69.8% | 64.0% | 80.2% |
| **Other Screenings** |  |  |  |  |  |  |  |
| Blood pressure | 96.3% | 97.4% | 95.1% | 95.4% | 93.6% | 92.2% | 97.0% |
| Cholesterol | 92.6% | 91.8% | 93.3% | 91.9% | 91.7% | 90.1% | 92.9% |

AIAN=American Indian or Alaska Native; NHPI=Native Hawaiian or Pacific Islander

^a^ Among females only

**Supplemental Table 5.** Prevalence and adjusted association between patient-clinician sex concordance and adherence to preventive services guidelines, overall. Results are weighted to be nationally representative and account for MEPS complex sample design.

|  | **Sex**  **Concordance** | **Sex**  **Discordance** | **PR (95% CI)^a^** |  |
| --- | --- | --- | --- | --- |
| **Vaccinations** |  |  |  |  |
| Influenza | 57.3% | 55.7% | 1.03 (1.00, 1.06) |  |
| Pneumococcal | 76.8% | 72.9% | 1.05 (1.02, 1.09) |  |
| Shingles | 52.3% | 49.9% | 1.05 (0.99, 1.11) |  |
| **Cancer Screening** |  |  |  |  |
| Breast | N/A^b^ | N/A^b^ | N/A^b^ |  |
| Cervical | N/A^b^ | N/A^b^ | N/A^b^ |  |
| Colorectal | 79.1% | 77.8% | 1.02 (0.99, 1.04) |  |
| **Other Screenings** |  |  |  |  |
| Blood Pressure | 92.7% | 92.6% | 1.01 (1.00, 1.01) |  |
| Cholesterol | 96.6% | 96.1% | 1.00 (0.99, 1.01) |  |
| Abbreviations: PR, prevalence ratio; CI, confidence interval; N/A, not analyzed  ^a^ Prevalence ratio (PR) is ratio of predicted marginal proportions calculated from logistic regression model adjusted for patient sex, race and/or ethnicity, age (treated as a restricted quadratic spline), highest education, family income (treated as a restricted quadratic spline), insurance status, self-reported chronic conditions (heart disease or stroke, diabetes, asthma, and cancer), self-reported general health, survey year, and clinician race and/or ethnicity  ^b^ Breast and cervical cancer screening guidelines only apply to females and was only analyzed in sex-stratified analyses | | | | |

**Supplemental Table 6.** Prevalence and association between patient-clinician race and/or ethnicity concordance and adherence to preventive services guidelines, overall. Results are weighted to be nationally representative and account for MEPS complex sample design.

|  | **Race and/or**  **ethnicity**  **concordance** | **Race and/or**  **ethnicity**  **discordance** | **PR (95% CI)^a^** |  |
| --- | --- | --- | --- | --- |
| **Vaccinations** |  |  |  |  |
| Influenza | 56.4% | 57.1% | 0.99 (0.94, 1.04) |  |
| Pneumococcal | 73.7% | 78.6% | 0.94 (0.89, 0.99) |  |
| Shingles | 50.4% | 54.0% | 0.93 (0.84, 1.04) |  |
| **Cancer Screening** |  |  |  |  |
| Breast^b^ | 82.7% | 83.1% | 1.00 (0.94, 1.06) |  |
| Cervical^b^ | 84.1% | 82.9% | 1.01 (0.97, 1.06) |  |
| Colorectal | 78.5% | 78.9% | 1.00 (0.96, 1.04) |  |
| **Other Screenings** |  |  |  |  |
| Blood pressure | 96.3% | 96.4% | 1.00 (0.99, 1.01) |  |
| Cholesterol | 92.7% | 92.4% | 1.00 (0.98, 1.03) |  |
| Abbreviations: PR, prevalence ratio; CI, confidence interval  ^a^ Prevalence ratio (PR) is ratio of predicted marginal proportions calculated from logistic regression model adjusted for patient sex, race and/or ethnicity, age (treated as a restricted quadratic spline), highest education, family income (treated as a restricted quadratic spline), insurance status, self-reported chronic conditions (heart disease or stroke, diabetes, asthma, and cancer), self-reported general health, survey year, and clinician sex  ^b^ Among females only | | | | |

**Supplemental Table 7.** Prevalence and association between patient-clinician race and/or ethnicity concordance and adherence to preventive services guidelines, among non-White participants. Results are weighted to be nationally representative and account for MEPS complex sample design.

|  | **Race and/or**  **ethnicity**  **concordance** | **Race and/or**  **ethnicity**  **discordance** | **PR (95% CI)^a^** |  |
| --- | --- | --- | --- | --- |
| **Vaccinations** |  |  |  |  |
| Influenza | 47.1% | 48.0% | 0.98 (0.86, 1.12) |  |
| Pneumococcal | 62.5% | 62.9% | 0.99 (0.82, 1.19) |  |
| Shingles | 31.8% | 39.2% | 0.81 (0.57, 1.17) |  |
| **Cancer Screening** |  |  |  |  |
| Breast^b^ | 79.3% | 84.2% | 0.94 (0.83, 1.06) |  |
| Cervical^b^ | 77.0% | 78.9% | 0.98 (0.88, 1.09) |  |
| Colorectal | 75.3% | 72.0% | 1.05 (0.95, 1.15) |  |
| **Other Screenings** |  |  |  |  |
| Blood pressure | 95.1% | 94.2% | 1.01 (0.98, 1.04) |  |
| Cholesterol | 94.0% | 90.8% | 1.04 (1.00, 1.07) |  |
| Abbreviations: PR, prevalence ratio; CI, confidence interval  ^a^ Prevalence ratio (PR) is ratio of predicted marginal proportions calculated from logistic regression model adjusted for patient sex, race and/or ethnicity, age (treated as a restricted quadratic spline), highest education, family income (treated as a restricted quadratic spline), insurance status, self-reported chronic conditions (heart disease or stroke, diabetes, asthma, and cancer), self-reported general health, survey year, and clinician sex  ^b^ Among females only | | | | |

**Supplemental Table 8.** Prevalence of adherence to preventive services guidelines, stratified by patient-clinician race and/or ethnicity concordance. Results are weighted to be nationally representative and account for MEPS complex sample design.

|  | **Asian** | | **Black** | | **Latino** | | **White** | |
| --- | --- | --- | --- | --- | --- | --- | --- | --- |
|  | **Race and/or**  **ethnicity**  **concordance** | **Race and/or**  **ethnicity**  **discordance** | **Race and/or**  **ethnicity**  **concordance** | **Race and/or**  **ethnicity**  **discordance** | **Race and/or**  **ethnicity**  **concordance** | **Race and/or**  **ethnicity**  **discordance** | **Race and/or**  **ethnicity**  **concordance** | **Race and/or**  **ethnicity**  **discordance** |
| **Vaccinations** |  |  |  |  |  |  |  |  |
| Influenza | 56.4% | 65.0% | 41.9% | 43.9% | 44.0% | 47.2% | 59.7% | 60.0% |
| Pneumococcal | 66.0% | 60.7% | 52.4% | 66.4% | 55.9% | 64.8% | 77.8% | 79.8% |
| Shingles | 36.9% | 51.1% | 34.4% | 31.5% | 28.5% | 37.1% | 54.5% | 55.7% |
| **Cancer Screening** |  |  |  |  |  |  |  |  |
| Breast^a^ | 82.3% | 84.1% | 84.6% | 84.3% | 84.7% | 81.0% | 82.3% | 85.3% |
| Cervical^a^ | 68.3% | 80.2% | 77.2% | 74.9% | 81.3% | 82.1% | 86.4% | 83.4% |
| Colorectal | 77.3% | 69.1% | 71.4% | 79.1% | 69.3% | 70.1% | 80.1% | 80.7% |
| **Other Screenings** |  |  |  |  |  |  |  |  |
| Blood pressure | 88.3% | 86.8% | 96.0% | 95.2% | 93.5% | 93.7% | 96.9% | 97.2% |
| Cholesterol | 90.0% | 83.8% | 91.4% | 92.0% | 93.2% | 90.9% | 92.7% | 94.1% |
| ^a^ Among females only | | | | | | | | |

**Supplemental Table 9.** Prevalence of adherence to preventive services guidelines, stratified by patient-clinician race and/or ethnicity concordance, among non-White participants. Results are weighted to be nationally representative and account for MEPS complex sample design.

|  | **Asian** | | | **Black** | | | **Latino** | | |
| --- | --- | --- | --- | --- | --- | --- | --- | --- | --- |
|  | **Race and/or**  **Ethnicity**  **Concordance** | **Race and/or Ethnicity Discordance,**  **Non-White** | **Race and/or Ethnicity Discordance,**  **White** | **Race and/or**  **Ethnicity**  **Concordance** | **Race and/or**  **Ethnicity**  **Discordance,**  **Non-White** | **Race and/or**  **Ethnicity**  **Discordance,**  **White** | **Race and/or**  **Ethnicity**  **Concordance** | **Race and/or**  **Ethnicity**  **Discordance,**  **Non-White** | **Race and/or**  **Ethnicity**  **Discordance,**  **White** |
| **Vaccinations** |  |  |  |  |  |  |  |  |  |
| Influenza | 56.3% | 61.4% | 66.2% | 41.9% | 43.1% | 44.3% | 44.0% | 47.2% | 47.2% |
| Pneumococcal | 66.0% | 58.4% | 61.6% | 52.4% | 62.3% | 68.2% | 55.8% | 61.3% | 67.0% |
| Shingles | 37.5% | 37.5% | 57.6% | 34.4% | 33.0% | 30.8% | 28.4% | 33.8% | 39.1% |
| **Cancer Screening** |  |  |  |  |  |  |  |  |  |
| Breast^a^ | 82.5% | 78.5% | 86.7% | 84.7% | 87.3% | 82.8% | 84.7% | 87.8% | 77.1% |
| Cervical^a^ | 68.2% | 84.3% | 78.7% | 77.2% | 75.1% | 74.9% | 81.3% | 82.8% | 81.6% |
| Colorectal | 77.4% | 58.6% | 73.5% | 71.5% | 84.0% | 76.9% | 69.3% | 64.5% | 73.4% |
| **Other Screening** |  |  |  |  |  |  |  |  |  |
| Blood pressure | 88.3% | 87.7% | 86.3% | 96.0% | 94.3% | 95.6% | 93.5% | 92.3% | 94.4% |
| Cholesterol | 90.0% | 84.8% | 83.4% | 91.4% | 90.1% | 92.9% | 93.2% | 90.0% | 91.3% |
| Note: Racial and/or ethnic concordance was categorized as 1) yes, 2) no, but clinician is non-White, and 3) no and clinician is White. Due to small population sizes and very low patient-clinician concordance, AIAN and NHPI patients were excluded from these analyses  ^a^ Among females only | | | | | | | | | |
